# Severe pneumocranium after gamma knife stereotactic radiosurgery for brain metastasis: A case report and literature review

**DOI:** 10.1097/MD.0000000000038464

**Published:** 2024-06-07

**Authors:** Paul J. Chen, Hung-Lin Lin

**Affiliations:** a Department of Neurosurgery, China Medical University Hospital, Taichung City, Taiwan (R.O.C.).

**Keywords:** brain metastasis, cerebrospinal fluid leakage, gamma knife stereotactic radiosurgery (GKRS), pneumocranium

## Abstract

**Rationale:**

Gamma knife stereotactic radiosurgery (GKRS) is a recognized safe and effective treatment for brain metastasis; however, some complications can present significant clinical challenges. This case report highlights a rare occurrence of cerebrospinal fluid (CSF) leakage and pneumocranium following GKRS, emphasizing the need for awareness and prompt management of these complications.

**Patient concerns:**

A 35-year-old male with a history of malignant neoplasm of the lip in 2015 and perineural spread of malignancy into the left cavernous sinus was treated with GKRS in 2017. The patient was admitted emergently 39 days after discharge due to persistent headache and dizziness.

**Diagnoses:**

Brain computed tomography (CT) revealed diffuse bilateral pneumocranium alongside an observation of CSF leakage.

**Interventions:**

A surgical procedure involving a left frontal-temporal craniotomy was performed to excise a residual skull base tumor and repair the dura, guided by a navigator system. The conclusive pathological assessment revealed the presence of squamous cell carcinoma markers.

**Outcomes:**

The patient exhibited excellent tolerance to the entire procedure and experienced a prompt and uneventful recovery process. After surgery, the symptoms alleviated and CSF leak stopped. The follow-up image showed the pneumocranium resolved.

**Lessons:**

Pneumocranium due to early-stage post-GKRS is uncommon. The rapid tumor shrinkage and timing of brain metastasis spreading through the dura can lead to CSF leak and pneumocranium. We reviewed current treatment options and presented a successful craniotomy-based dura repair case.

## 1. Introduction

Brain metastases are adults’ most common type of intracranial neoplasm.^[1,2]^ Brain metastases develop when malignant cells from a primary site migrate to the brain. While brain metastases can stem from diverse locations and cancer types, those originating in the breast, colon, kidney, lung, and melanoma generally possess an elevated likelihood of inducing brain metastases.^[3]^

Brain tumor surgery presents the challenge of removing as many tumors as possible while minimizing damage to healthy brain tissue.^[4,5]^ Excision surgery is considered the most effective treatment for solid tumors.^[6]^ However, craniotomy may not consistently achieve the desired outcome ideally.^[7]^ As a result, advanced technologies have been developed for brain cancer treatment.

Gamma knife stereotactic radiosurgery (GKRS) is an advanced and precise therapeutic approach that has demonstrated efficacy in managing various intracranial conditions, including brain tumors, arteriovenous malformations, and trigeminal neuralgia.^[8–10]^ This noninvasive technique delivers highly focused gamma rays to specific intracranial targets, achieving remarkable precision while sparing surrounding healthy brain tissue.^[8]^ In spite of its well-established advantages and overall safety profile, there are documented instances of complications reported after undergoing GKRS.^[11,12]^

The potential complications after GKRS can vary depending on the specific condition being treated and the location of the targeted area within the brain.^[11,13–15]^ Possible resolvable complications include swelling, edema, nausea and vomiting, headache, fatigue, hair loss, and visual or auditory disturbances. However, more severe complications such as seizures, radiation necrosis, radiation-induced tumors, and pneumocranium require immediate treatment.

Here, we present a case of severe pneumocranium subsequent to GKRS for brain metastasis in a patient. Additionally, a comprehensive literature review was conducted to draw a comparison with the current case.

## 2. Case report

The patient was a 35-year-old man with a previous diagnosis of left lip cancer, classified explicitly as pT2N2bM0. In 2015 to 2016, the patient underwent a composite resection for left upper lip cancer, which involved sacrificing the affected skin, performing an inferior maxillectomy, and a marginal mandibulectomy. Additionally, the patient underwent a left modified radical neck dissection and a right selective neck dissection. Following the surgery, free flap reconstruction was performed. Since the procedure, the patient has regularly attended follow-up appointments at our ear, nose, and throat outpatient department (OPD).

The patient had been experiencing left ptosis (drooping eyelid) and eye protrusion for 2 weeks before seeking medical advice. He sought medical attention at the oncology OPD and was subsequently referred to the Neurosurgery OPD for comprehensive assessment in 2017. Neurological examination revealed the Glasgow Coma Scale score of E4V5M6, and the pupil sizes were measured at 4.0+ for the right eye and 3.5− for the left eye. Muscle power was found to be intact in all tested muscles. The patient mentioned experiencing a similar episode about 2 years ago, during which he had recovered well.

**Figure 1.**
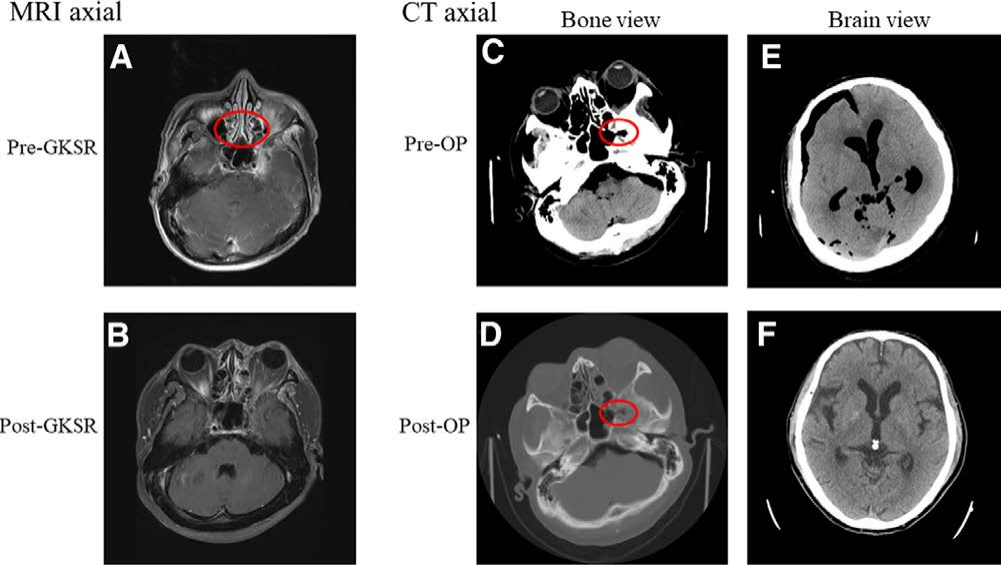
MRI and CT images at different times during the therapy. MRI image was taken at pre- and post-GKRS (A, B). Bone (C, D) and brain (E, F) view of CT pre- and after left frontotemporal (F–T) craniotomy. CT = computed tomography, GKRS = gamma knife stereotactic radiosurgery, MRI = magnetic resonance imaging.

Considering the clinical presentation, our primary impressions include left ptosis, which could suggest brain metastasis or carotid-cavernous fistula. The patient was admitted to our medical ward as part of the evaluation and management process. Magnetic resonance imaging and Orbit computed tomography (CT) scan showed evidence of perineural spread of malignancy originating from the left skull base, extending into the cavernous sinus, and causing bony destruction of the left sphenoid wing (Fig. 1A and B).

Upon admission, GKRS was promptly scheduled for the patient on the second day. During the procedure, we identified a left skull base tumor with invasion into the cavernous sinus, measuring approximately 21.3 cc. The treatment plan involved applying a 50% marginal dose of 16 Gy, with a mean dose of 19.6 Gy. We used 18 mm and 14 mm collimators with plugs to perform the radiosurgery. The entire procedure lasted approximately 1.5 hours. Following the radiosurgery, the patient experienced eye swelling on the first day after the operation. In response, we prescribed dexamethasone to manage the swelling and provided pain control measures. Subsequently, by the fourth day of admission, the patient condition had shown significant improvement, allowing for discharge from the hospital with plans for follow-up at our OPD.

At 39 days following discharge, the patient was urgently taken to the emergency department (ED) due to ongoing complaints of persistent headache and dizziness. Physical examination revealed increased intracranial pressure and meningeal signs, indicating possible involvement of the protective membrane covering the brain (dura). A Brain CT scan showed severe pneumocranium (Fig. 1C–F). Based on these findings, the provisional diagnosis was a cerebrospinal fluid (CSF) leak with pneumocranium.

**Figure 2.**
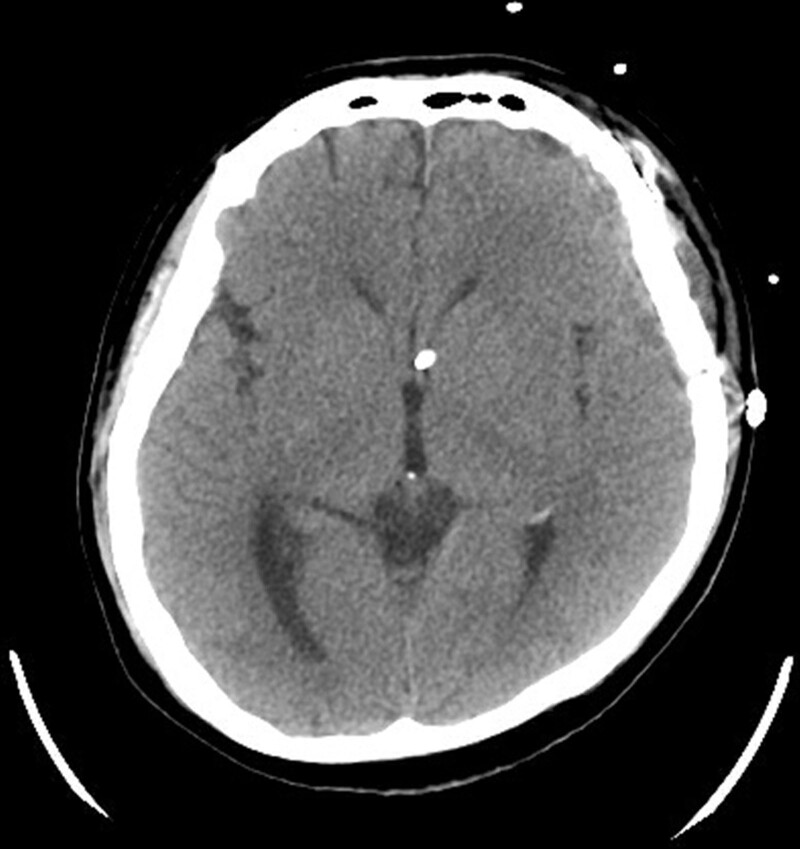
CT image on the 11th day following craniotomy. CT = computed tomography.

In response to the critical condition, the patient was promptly admitted for emergent surgery to repair the dura. The surgical procedure performed was a left frontotemporal craniotomy, removing a tumor at the skull base. In immunohistochemical analysis, the specimen showed positive for cytokeratin and p40, marker of squamous cell carcinoma. The tumor was situated in the left temporal region and had caused a defect in the dura, extending further into the lateral sphenoid sinus. The surgical approach was guided using a navigator, and an external ventricular drain was also utilized during the operation. After surgery, the headache and dizziness symptoms were alleviated, and the CSF leak stopped. The follow-up CT image by the eleventh day postoperatively showed the pneumocranium was no longer present (Fig. 2). The patient was discharged with plans for follow-up at our OPD.

## 3. Discussion

A 35-year-old man with a history of left lip cancer was diagnosed with brain metastasis at the skull base and treated by GKRS. The patient recovered well and was released. The patient was admitted 39 days after discharge with persistent headache and dizziness. A Brain CT scan showed severe pneumocranium, likely caused by CSF leakage. The patient received emergent surgery to repair the dura. After the surgery, the symptoms of headache and dizziness were alleviated, the CSF leak stopped, and pneumocranium resolved. Close monitoring was maintained.

CSF fills the cranial cavity to protect the brain and spinal cord from damage from the outside. When the dura mater, the outermost tissue surrounding the CSF, is broken, CSF may leak from the cranial space. CSF leakage is a known but infrequent complication in conventional transsphenoidal surgery, with reported incidence rates ranging from 0.4% to 9%.^[16,17]^ Conversely, CSF leakage had been rarely documented in noninvasive surgical procedures like GKRS. Kim et al^[18]^ reported a case of CSF leakage occurring 4 months after GKRS in a patient with skull base metastasis.

Similarly, Ogawa et al^[19]^ described a case of CSF leakage ten years after a combination of transsphenoidal surgery and GKRS in a patient with prolactin-secreting pituitary adenoma. Two other patients with pituitary adenoma underwent transsphenoidal surgery followed by GKRS and experienced CSF leakage 2 and 7 years after GKRS, respectively.^[20]^ Another case of CSF leakage was reported in a cohort of 32 patients with malignant skull base tumors.^[21]^ Although the incidence of CSF leakage after GKRS is very low, as demonstrated in these cases, it remains a significant complication that requires careful consideration.

**Figure 3.**
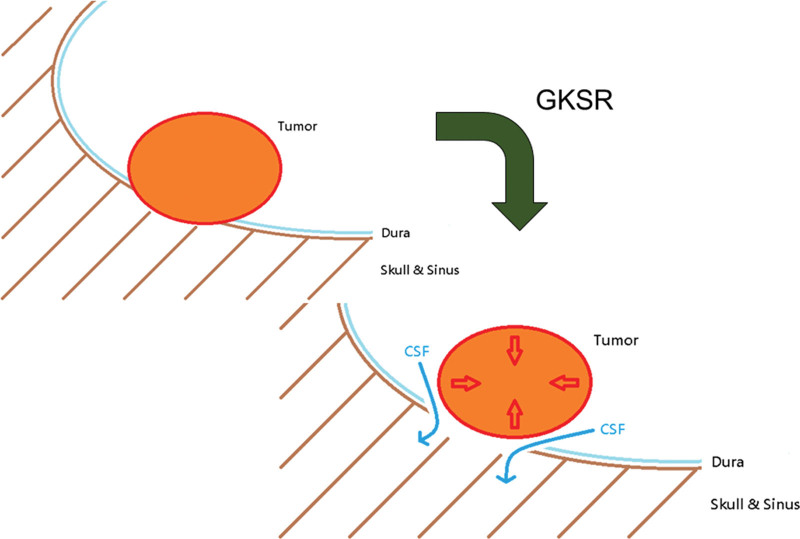
The proposed scenario of pneumocranium formation after GKRS. GKRS = gamma knife stereotactic radiosurgery.

When air enters the cranial space, it is called pneumocephalus. About 25% of patients with pneumocephalus were caused by factors other than trauma.^[22,23]^ One of these factors was a tumor at the skull base. Metastasis cancer corrodes or invades the surrounding tissue. Since the dura thickness in the skull base was relatively thinner than other parts,^[24]^ it was easier for cancer cells to breach the CSF leak-proof mechanism in the skull base. When the tumor is shrunken after surgery, the CSF may leak from those cracks made by cancer, and air may be drawn into the cranial space via the inverted soda bottle mechanism^[16–18]^ or the ball valve mechanism.^[25–28]^ This scenario was described in several clinical case reports (Fig. 3).^[29,30]^

Pollock et al^[31]^ reported that the size of nonfunctioning pituitary adenomas decreased in 60% of patients after gamma knife surgery. The mean volume reduction in vestibular schwannomas after GKRS was 31%.^[32]^ This indicated that although the primary goal of GKRS was suppressing tumor growth, the tumor did shrink after GKRS. Once the tumor shrank and exposed the defects caused by tumor erosion in the dura, CSF may start leaking immediately. Although rare, this had been reported after dopaminergic agonists treated prolactinoma,^[33]^ after spine tumor resection,^[34]^ and after GKRS.^[32]^ These reports indicated that the shrink-and-leak scenario might be the mechanism behind observed pneumocranium after GKRS for brain metastasis.

## 4. Limitations

Our study is subject to several limitations. Firstly, being a single case report, the findings may not be generalizable. Secondly, the proposed mechanism for the development of pneumocranium is speculative. Third, the relatively short follow-up period may not fully capture long-term outcomes or recurrent complications. These limitations highlight the need for more extensive studies to validate and expand upon these findings.

## 5. Conclusion

Although rare, pneumocranium can occur after GKRS. If a patient complains about new or worsening symptoms, pneumocranium should be suspected and checked immediately after therapy.

## Author contributions

**Conceptualization:** Paul J. Chen.

**Data curation:** Paul J. Chen, Hung-Lin Lin.

**Formal analysis:** Paul J. Chen, Hung-Lin Lin.

**Investigation:** Hung-Lin Lin.

**Methodology:** Hung-Lin Lin.

**Writing – original draft:** Paul J. Chen.

**Writing – review & editing:** Paul J. Chen.

## References

[1] Saha A Ghosh SK Roy C. Demographic and clinical profile of patients with brain metastases: a retrospective study. Asian J Neurosurg. 2013;8:157–61. 24403959 10.4103/1793-5482.121688 PMC3877503

[2] Achrol AS Rennert RC Anders C. Brain metastases. Nat Rev Dis Primers. 2019;5:5. 30655533 10.1038/s41572-018-0055-y

[3] Boire A Brastianos PK Garzia L. Brain metastasis. Nat Rev Cancer. 2020;20:4–11. 31780784 10.1038/s41568-019-0220-y

[4] Lakomkin N Hadjipanayis CG. The use of spectroscopy handheld tools in brain tumor surgery: current evidence and techniques. Front Surg. 2019;6:30. 31192217 10.3389/fsurg.2019.00030 PMC6548876

[5] Królikowska A Filipska-Blejder K Jabłońska R. Quality of life after surgical treatment of brain tumors. J Clin Med. 2022;11:3733. 35807017 10.3390/jcm11133733 PMC9267496

[6] Benjamin DJ. The efficacy of surgical treatment of cancer - 20 years later. Med Hypotheses. 2014;82:412–20. 24480434 10.1016/j.mehy.2014.01.004

[7] Subbarao BS Fernández-de Thomas RJ Eapen BC. Post craniotomy headache. StatPearls. Treasure Island (FL) ineligible companies. Disclosure: Ricardo Fernández-de Thomas declares no relevant financial relationships with ineligible companies. Disclosure: Blessen Eapen declares no relevant financial relationships with ineligible companies.: StatPearls Publishing Copyright © 2023, StatPearls Publishing LLC.; 2023.

[8] Desai R Rich KM. Therapeutic role of gamma knife stereotactic radiosurgery in neuro-oncology. Mo Med. 2020;117:33–8. 32158047 PMC7023953

[9] Suh JH. Stereotactic radiosurgery for the management of brain metastases. N Engl J Med. 2010;362:1119–27. 20335588 10.1056/NEJMct0806951

[10] Chang EL Selek U Hassenbusch SJ 3rd. Outcome variation among “radioresistant” brain metastases treated with stereotactic radiosurgery. Neurosurgery. 2005;56:936–45; discussion 936. 15854241

[11] Barisano G Bergamaschi S Acharya J. Complications of radiotherapy and radiosurgery in the brain and spine. Neurographics (2011). 2018;8:167–87. 35388375 10.3174/ng.1700066 PMC8981962

[12] Vachhrajani S Fawaz C Mathieu D. Complications of Gamma Knife surgery: an early report from 2 Canadian centers. J Neurosurg. 2008;109(Suppl):2–7. 19123881 10.3171/JNS/2008/109/12/S2

[13] Chao ST Thakkar VV Barnett GH. Prospective study of the short-term adverse effects of gamma knife radiosurgery. Technol Cancer Res Treat. 2012;11:117–22. 22335405 10.7785/tcrt.2012.500240

[14] Chin LS Lazio BE Biggins T. Acute complications following gamma knife radiosurgery are rare. Surg Neurol. 2000;53:498–502; discussion 502. 10874151 10.1016/s0090-3019(00)00219-6

[15] Kano H Flickinger JC Tonetti D. Estimating the risks of adverse radiation effects after gamma knife radiosurgery for arteriovenous malformations. Stroke. 2017;48:84–90. 27899758 10.1161/STROKEAHA.116.014825

[16] Tamasauskas A Sinkūnas K Draf W. Management of cerebrospinal fluid leak after surgical removal of pituitary adenomas. Medicina (Kaunas). 2008;44:302–7. 18469507

[17] Buchfelder M Schlaffer S. Surgical treatment of pituitary tumours. Best Pract Res Clin Endocrinol Metab. 2009;23:677–92. 19945031 10.1016/j.beem.2009.05.002

[18] Kim CH Chung SK Dhong HJ. Cerebrospinal fluid leakage after gamma knife radiosurgery for skull base metastasis from renal cell carcinoma: a case report. Laryngoscope. 2008;118:1925–7. 18797420 10.1097/MLG.0b013e3181820171

[19] Ogawa Y Tominaga T. Delayed cerebrospinal fluid leakage 10 years after transsphenoidal surgery and gamma knife surgery - case report. Neurol Med Chir (Tokyo). 2007;47:483–5. 17965568 10.2176/nmc.47.483

[20] Perry A Graffeo CS Copeland WR 3rd. Delayed cerebrospinal fluid rhinorrhea after gamma knife radiosurgery with or without preceding transsphenoidal resection for pituitary pathology. World Neurosurg. 2017;100:201–7. 28089836 10.1016/j.wneu.2017.01.001

[21] Miller RC Foote RL Coffey RJ. The role of stereotactic radiosurgery in the treatment of malignant skull base tumors. Int J Radiat Oncol Biol Phys. 1997;39:977–81. 9392534 10.1016/s0360-3016(97)00377-5

[22] Karavelioglu E Eser O Haktanir A. Pneumocephalus and pneumorrhachis after spinal surgery: case report and review of the literature. Neurol Med Chir (Tokyo). 2014;54:405–7. 24305016 10.2176/nmc.cr2013-0118 PMC4533435

[23] Gorissen Z Hakvoort K van den Boogaart M. A rare and life-threatening, but reversible, complication after penetrating lumbar injury. Acta Neurochir (Wien). 2019;161:361–5. 30652201 10.1007/s00701-018-03796-y PMC6373275

[24] Willatt DJ Yung MW Helliwell TR. A correlation of the surgical anatomy of the dura to head and neck surgery. Arch Otorhinolaryngol. 1987;243:403–6. 3566624 10.1007/BF00464652

[25] Ihab Z. Pneumocephalus after surgical evacuation of chronic subdural hematoma: Is it a serious complication? Asian J Neurosurg. 2012;7:66–74. 22870154 10.4103/1793-5482.98647 PMC3410163

[26] Sweni S Senthilkumaran S Balamurugan N. Tension pneumocephalus: a case report with review of literature. Emerg Radiol. 2013;20:573–8. 23748929 10.1007/s10140-013-1135-7

[27] Yun JH Kim YJ Yoo DS. Diffuse pneumocephalus: a rare complication of spinal surgery. J Korean Neurosurg Soc. 2010;48:288–90. 21082062 10.3340/jkns.2010.48.3.288 PMC2966736

[28] Solomiichuk VO Lebed VO Drizhdov KI. Posttraumatic delayed subdural tension pneumocephalus. Surg Neurol Int. 2013;4:37. 23607059 10.4103/2152-7806.109537 PMC3622390

[29] Risso A Zoia C Gianformaggio C. Tension pneumocephalus secondary to osteoradionecrosis of the clivus. Rep Pract Oncol Radiother. 2016;21:71–5. 26900361 10.1016/j.rpor.2015.05.007 PMC4716398

[30] Jimenez-Jimenez E Martí SS Villas MV. Tension pneumocephalus related to radiotherapy for nasopharyngeal carcinoma. Case Rep Oncol Med. 2014;2014:327380. 25210637 10.1155/2014/327380 PMC4158148

[31] Pollock BE Cochran J Natt N. Gamma knife radiosurgery for patients with nonfunctioning pituitary adenomas: results from a 15-year experience. Int J Radiat Oncol Biol Phys. 2008;70:1325–9. 18029107 10.1016/j.ijrobp.2007.08.018

[32] Nagano O Serizawa T Higuchi Y. Tumor shrinkage of vestibular schwannomas after Gamma Knife surgery: results after more than 5 years of follow-up. J Neurosurg. 2010;113(Suppl):122–27. 21222292

[33] Pechman A Manavalan A Kishore P. Cerebrospinal fluid leak after medical management of prolactinoma. J Endocr Soc. 2021;5:A576–A576.

[34] Barber SM Fridley JS Konakondla S. Cerebrospinal fluid leaks after spine tumor resection: avoidance, recognition and management. Ann Transl Med. 2019;7:217. 31297382 10.21037/atm.2019.01.04 PMC6595203

